# Intratracheally administered LNA gapmer antisense oligonucleotides induce robust gene silencing in mouse lung fibroblasts

**DOI:** 10.1093/nar/gkac630

**Published:** 2022-08-03

**Authors:** Minwook Shin, Io Long Chan, Yuming Cao, Alisha M Gruntman, Jonathan Lee, Jacquelyn Sousa, Tomás C Rodríguez, Dimas Echeverria, Gitali Devi, Alexandre J Debacker, Michael P Moazami, Pranathi Meda Krishnamurthy, Julia M Rembetsy-Brown, Karen Kelly, Onur Yukselen, Elisa Donnard, Teagan J Parsons, Anastasia Khvorova, Erik J Sontheimer, René Maehr, Manuel Garber, Jonathan K Watts

**Affiliations:** RNA Therapeutics Institute, University of Massachusetts Chan Medical School, Worcester, MA 01605, USA; RNA Therapeutics Institute, University of Massachusetts Chan Medical School, Worcester, MA 01605, USA; Program in Bioinformatics and Integrative Biology, University of Massachusetts Chan Medical School, Worcester, MA 01605, USA; Horae Gene Therapy Center, University of Massachusetts Chan Medical School, Worcester, MA 01605, USA; Department of Pediatrics, University of Massachusetts Chan Medical School, Worcester, MA 01605, USA; Department of Clinical Sciences, Cummings School of Veterinary Medicine at Tufts University, N. Grafton, MA 01536, USA; RNA Therapeutics Institute, University of Massachusetts Chan Medical School, Worcester, MA 01605, USA; RNA Therapeutics Institute, University of Massachusetts Chan Medical School, Worcester, MA 01605, USA; RNA Therapeutics Institute, University of Massachusetts Chan Medical School, Worcester, MA 01605, USA; RNA Therapeutics Institute, University of Massachusetts Chan Medical School, Worcester, MA 01605, USA; RNA Therapeutics Institute, University of Massachusetts Chan Medical School, Worcester, MA 01605, USA; RNA Therapeutics Institute, University of Massachusetts Chan Medical School, Worcester, MA 01605, USA; RNA Therapeutics Institute, University of Massachusetts Chan Medical School, Worcester, MA 01605, USA; RNA Therapeutics Institute, University of Massachusetts Chan Medical School, Worcester, MA 01605, USA; RNA Therapeutics Institute, University of Massachusetts Chan Medical School, Worcester, MA 01605, USA; RNA Therapeutics Institute, University of Massachusetts Chan Medical School, Worcester, MA 01605, USA; Program in Bioinformatics and Integrative Biology, University of Massachusetts Chan Medical School, Worcester, MA 01605, USA; Program in Bioinformatics and Integrative Biology, University of Massachusetts Chan Medical School, Worcester, MA 01605, USA; Program in Molecular Medicine, University of Massachusetts Chan Medical School, Worcester, MA 01605, USA; Diabetes Center of Excellence, University of Massachusetts Chan Medical School, Worcester, MA 01605, USA; RNA Therapeutics Institute, University of Massachusetts Chan Medical School, Worcester, MA 01605, USA; Program in Molecular Medicine, University of Massachusetts Chan Medical School, Worcester, MA 01605, USA; RNA Therapeutics Institute, University of Massachusetts Chan Medical School, Worcester, MA 01605, USA; Program in Molecular Medicine, University of Massachusetts Chan Medical School, Worcester, MA 01605, USA; Li Weibo Institute for Rare Diseases Research, University of Massachusetts Chan Medical School, Worcester, MA 01605, USA; Program in Molecular Medicine, University of Massachusetts Chan Medical School, Worcester, MA 01605, USA; Diabetes Center of Excellence, University of Massachusetts Chan Medical School, Worcester, MA 01605, USA; Program in Bioinformatics and Integrative Biology, University of Massachusetts Chan Medical School, Worcester, MA 01605, USA; Program in Molecular Medicine, University of Massachusetts Chan Medical School, Worcester, MA 01605, USA; RNA Therapeutics Institute, University of Massachusetts Chan Medical School, Worcester, MA 01605, USA; Li Weibo Institute for Rare Diseases Research, University of Massachusetts Chan Medical School, Worcester, MA 01605, USA; Department of Biochemistry and Molecular Biotechnology, University of Massachusetts Chan Medical School, Worcester, MA 01605, USA

## Abstract

The lung is a complex organ with various cell types having distinct roles. Antisense oligonucleotides (ASOs) have been studied in the lung, but it has been challenging to determine their effectiveness in each cell type due to the lack of appropriate analytical methods. We employed three distinct approaches to study silencing efficacy within different cell types. First, we used lineage markers to identify cell types in flow cytometry, and simultaneously measured ASO-induced silencing of cell-surface proteins CD47 or CD98. Second, we applied single-cell RNA sequencing (scRNA-seq) to measure silencing efficacy in distinct cell types; to the best of our knowledge, this is the first time scRNA-seq has been applied to measure the efficacy of oligonucleotide therapeutics. In both approaches, fibroblasts were the most susceptible to locally delivered ASOs, with significant silencing also in endothelial cells. Third, we confirmed that the robust silencing in fibroblasts is broadly applicable by silencing two targets expressed mainly in fibroblasts, *Mfap4* and *Adam33*. Across independent approaches, we demonstrate that intratracheally administered LNA gapmer ASOs robustly induce gene silencing in lung fibroblasts. ASO-induced gene silencing in fibroblasts was durable, lasting 4–8 weeks after a single dose. Thus, lung fibroblasts are well aligned with ASOs as therapeutics.

## INTRODUCTION

Gapmer antisense oligonucleotide (ASO) therapeutics are typically 16–20 nucleotides in length and consist of chemically modified nucleotides flanking a central section of DNA, linked mainly by phosphorothioate linkages (1,2). When the gapmer ASO specifically binds to the target RNA sequence via Watson–Crick base pairing, the central DNA region forms a DNA/RNA hybrid, eliciting cleavage of the RNA strand by ribonuclease H1 (3). Chemically modified nucleotides in the flanking regions improve therapeutic properties by increasing resistance to nucleases that contribute to the stability of therapeutics and modulating binding affinity for target RNA (4,5). Phosphorothioate linkages between nucleotides increase nuclease resistance and, at the same time, improve delivery into cells via protein binding (6). Locked nucleic acid (LNA) and its methylated analogue 2′,4′-constrained ethyl bicyclic nucleic acid (cEt) are among several chemical modifications that have been used to enhance the therapeutic properties of ASOs by increasing binding affinity to target RNA (7,8). Research and development of ASO therapeutics, including gapmers, is actively underway to treat diseases in various target tissues, with drugs approved for use in the liver, brain and eye (9–11).

Lung diseases, including asthma, chronic obstructive pulmonary disease, lung cancer, pulmonary fibrosis and cystic fibrosis (CF), represent significant unmet clinical needs (12,13). The lung has been considered a target organ for ASO therapeutics (14). Because of the unique nature of the lung, which is both exposed to the atmosphere and heavily vascularized, ASOs can be administered to the lung locally (via inhalation) or systemically (via injection) (15,16). Nevertheless, the lung is highly immunogenic, protected by mucus layers, and cellularly complex (15,17). ASOs have demonstrated their therapeutic potential for lung disease in various mouse models. Oropharyngeal instillation of cEt gapmer targeting Notch signaling pathway components *Notch2* or *Jag1* successfully prevented house dust mite-induced murine allergic asthma model (18). Similarly, a cEt gapmer targeting the sodium channel *ENaC* showed silencing in animals and advanced to clinical trials to treat CF (19,20). A 2′-*O*-methyl (2′-*O*-Me) gapmer targeting *Periostin* administered intranasally ameliorated bleomycin-induced lung fibrosis in a mouse model (21). LNA gapmers delivered to the murine lungs have been deployed to silence genes including *Scarb1* and *TGF-β2* (22,23).

ASOs have entered clinical development for lung diseases (24), but have failed to reach approval so far. We mention here two recent examples, both targeting airway epithelia, which demonstrated promise but were recently discontinued. The cEt gapmer ION-827359 (IONIS-ENaC-2.5Rx), developed by Ionis Pharmaceuticals, targets *ENaC* mRNA by ribonuclease H1 mediated cleavage and degradation. It showed reduced *ENaC* mRNA in airways and was well-tolerated in a human phase-1/2a clinical trial (NCT03647228) to treat CF (25), but its clinical development was stopped in 2021 due to undisclosed findings from a long-term preclinical toxicology study (Ionis Press Release, published at https://globalgenes.org/2021/05/10/ionis-stops-development-of-inhaled-antisense-therapy-for-cf/). ProQR therapeutics developed a 2′-*O*-Me and phosphorothioate steric block ASO, eluforsen (QR-010), which binds to *CFTR-F508del* mRNA and restores CFTR protein function by an unknown mechanism. Phase-1b and 2 clinical trials (NCT02564354, NCT02532764) showed no safety concerns and potential improvement in lung function (26,27), but its development was later terminated.

Lung function requires a complex tissue structure with diverse cell types, including epithelial cells, endothelial cells, leukocytes, and fibroblasts (28). Therapeutic approaches to treat lung disease likely require intervention in specific cell populations. Methods for analysing epithelial cells, endothelial cells, and leukocytes are relatively well defined, and previous research showed moderate target gene silencing in epithelial cells and leukocytes by administration of gapmer ASO (29,30), but fibroblasts have received little attention. An obstacle to analysing lung fibroblasts is the high degree of cellular heterogeneity depending on the status of the lungs in a physiological or pathological context (31–33).

Fibroblasts in the lung play a crucial role in development, homeostasis, injury response, and damage recovery, and should be carefully considered for treating lung disease (34,35). For example, increased number and aberrantly activated myofibroblasts are major components of the pathogenesis of fibrotic lung diseases such as idiopathic pulmonary fibrosis (36,37). But a comprehensive analysis method for lung fibroblasts has been lacking. A recently developed method to explore cell-type-specific biology is single-cell RNA sequencing (scRNA-seq), in which cells can be clustered into subpopulations by gene expression features consistent with the specific cell type (38,39). As such, a complex cell population is characterized by concurrent scanning of 10^3^ to 10^6^ transcriptomes per cell (40). However, analysis of the efficacy of target gene silencing by ASOs using scRNA-seq technology has not been reported.

In the present study, using flow cytometry and scRNA-seq analysis, we found that lung fibroblasts are the most susceptible target cell population for locally delivered LNA gapmers through intratracheal administration. We confirm that fibroblasts are an ideal cell population for ASO therapeutics by demonstrating robust ASO-mediated silencing of multiple target genes in vivo.

## MATERIALS AND METHODS

### Synthesis of ASOs

ASOs were synthesized using a Dr. Oligo 48 synthesizer (Biolytic Lab Performance, Fremont, CA, USA) with Unylinker controlled-pore glass (CPG) supports (ChemGenes, Burlington, MA, USA) and standard detritylation and capping reagents as described previously (41). Activation was achieved with 5-benzylmercaptotetrazole (0.25 M in acetonitrile, ChemGenes), oxidation involved the use of iodine (0.05 M in a 9:1 mixture of pyridine:water, ChemGenes), and sulfurization involved the use of 3-((dimethylamino-methylidene)amino)-3H-1,2,4-dithiazole-3-thione (DDTT) (0.1 M, ChemGenes). Anhydrous acetonitrile (MilliporeSigma, Burlington, MA, USA) was used to dissolve the DNA, 2′-*O*-methoxyethyl (2′-*O*-MOE), and LNA phosphoramidites (ChemGenes) to a final concentration of 0.15 M immediately before use, except for LNA 5-methyl C phosphoramidite which was dissolved in tetrahydrofuran (THF):acetonitrile (v:v = 3:1). DNA phosphoramidites and 2′-*O*-MOE/LNA phosphoramidites were coupled for 4 and 10 min, respectively.

The deprotection and cleavage from the CPG support were performed with concentrated NH_4_OH at 55°C for 16 h. After evaporation using a centrifugal evaporator (Eppendorf, Hamburg, Germany), the ASOs were resuspended in nuclease-free water (Thermo Fisher Scientific, Waltham, MA, USA). The resuspended ASOs were purified with reversed-phase high-performance liquid chromatography (RP-HPLC, Agilent, Santa Clara, CA, USA), then desalted using Amicon Ultra centrifugal filters (MilliporeSigma). All ASOs were analyzed using electrospray ionization quadrupole time-of-flight LC-MS (Agilent) in negative ionization mode. We observed synthesized oligos to be a clean single peak in the LC trace, and purity was higher than 85% in the mass analysis. Finally, the ASOs were dissolved in phosphate-buffered saline (PBS, pH 7.4, Thermo Fisher Scientific) at the desired concentration for animal administration. The sequences of the gapmer ASOs are shown in Table 1 and Supplementary Table S1.

**Table 1.** Gapmers ASOs used in this study. dN = DNA, +N = LNA, uN = 2′-*O*-MOE, d5C = 5-methyl-deoxycytosine. All internucleotide linkages were phosphorothioate

Target	Oligo name	Sequence (5′-3′)	Calculated mass	Observed mass
NTC	NTC_1	+A+A+CdAd5CdGdTd5CdTdAdTdA+C+G+C	4984.1	4983.6
	NTC_2	+A+T+TdTdTdAdTdTd5CdGdGdA+G+C+T	4994.0	4994.5
*Malat1*	Malat1_1	uGuGuGuUuCdAdGdCdTdGdCdCdAdAdTuGuCuUuAuG	7232.1	7232.0
	Malat1_2	+G+G+TdCdAdGdCdTdGdCdCdA+A+T+G	4985.9	4985.5
	Malat1_3	+C+T+AdGdTdTd5CdAd5CdTdGdAdA+T+G+C	5321.3	5320.5
*Cd47*	Cd47_4	+G+T+Gd5CdTdTdGdGd5CdGdAdGdT+C+T+C	5369.3	5368.5
*Cd98*	Cd98_6	+A+C+CdGdGd5Cd5Cd5CdGdAdAdTd5C+T+C+G	5334.4	5334.0
*Mfap4*	Mfap_4	+C+C+AdTdTdGdGdGd5Cd5Cd5CdAdA+T+T+G	5336.3	5337.3
*Adam33*	Adam33_1	+T+A+AdGd5CdTd5CdAdGdAdGdT+T+C+G	5027.1	5027.6

### Animal experiments

All animal procedures were conducted according to the Institutional Animal Care and Use Committee (IACUC) protocols of the University of Massachusetts Chan Medical School (IACUC protocol A-2551). 8-week-old female FVB mice (Charles River Laboratories, Wilmington, MA, USA) were administered 50 μl of PBS or gapmer ASO by intratracheal instillation as previously described (42). Mice were euthanized 2 days to 8 weeks after dosing, and tissues were collected. For RT-qPCR analysis of silencing efficacy or quantification of LNA gapmer, tissue samples were snap-frozen in liquid nitrogen and stored at -80°C until the further procedure. For flow cytometry analysis or scRNA-seq, mice lungs were immediately analyzed after collection.

### Flow cytometry analysis and sorting

Mouse lung was collected and stored into MACS Tissue Storage Solution (Miltenyi Biotec, Bergisch Gladbach, Germany) on ice while collecting lungs from multiple mice. An enzymatic dissociation solution was prepared with 100 μl of Enzyme D, 15 μl of Enzyme A, 62.5 μl of Enzyme P (Miltenyi Biotec, Skeletal Muscle Dissociation Kit), 250 U ml^–1^ Collagenase IV (Worthington, Lakewood, NJ, USA), and 2.3 ml of Dulbecco's Modified Eagle Medium (DMEM, MilliporeSigma). Collected mouse lung was dissociated to single-cell suspension using gentleMACS C Tubes (Miltenyi Biotec) and a gentleMACS Octo Dissociator (Miltenyi Biotec) with the enzymatic dissociation solution under the following conditions: running the gentleMACS program h_tumor_01, incubation at 37°C for 30 min, running the gentleMACS program h_tumor_01 a second time followed by incubation at 37°C for 30 min, and running the gentleMACS program m_lung_02. Dissociated cells were filtered through a 70-μm strainer and centrifuged at 500 × g at 4°C for 10 min. After discarding the supernatant, red blood cells (RBCs) were lysed using RBC lysis buffer (155 mM NH_4_Cl, 12 mM NaHCO_3_, 0.1 mM EDTA in distilled water), followed by washing with DMEM (500 × g, 4°C, 10 min). Next, the cell pellet was suspended in a flow cytometry buffer composed of 0.5% BSA and 2 mM EDTA in DMEM without phenol red (Thermo Fisher Scientific).

For flow cytometric sorting, lung cells were stained at 4°C for 30 min with the following antibodies: VioGreen-conjugated CD45 antibody (Miltenyi Biotec, clone REA737), APC-conjugated CD31 antibody (Miltenyi Biotec, clone REA784), FITC-conjugated CD326 antibody (Miltenyi Biotec, clone REA977) and PE-Vio770-conjugated CD140a antibody (Miltenyi Biotec, clone REA637). Cells were then washed twice with 700 μl of the flow cytometry buffer and resuspended in the flow cytometry buffer containing 1 μM SYTOX Blue (Thermo Fisher Scientific). Stained cells were sorted using BD FACSAria Fusion flow cytometer (BD Biosciences, Franklin Lakes, NJ, USA).

For measuring CD47 expression level, cells were stained as described above with the following antibodies: VioGreen-conjugated CD45 antibody, APC-conjugated CD31 antibody, FITC-conjugated CD326 antibody, PE-Vio770-conjugated CD140a antibody, and PE-conjugated CD47 antibody (Miltenyi Biotec, clone REA170). In the case of measuring CD98 or CD29 expression level, a PE-conjugated CD98 antibody (Miltenyi Biotec, clone REA861) or PE-conjugated CD29 antibody (Miltenyi Biotec, clone REA1074) was used instead of a PE-conjugated CD47 antibody, respectively. Stained cells were analyzed using a MACSQuant VYB flow cytometer (Miltenyi Biotec), and data were analyzed using FlowJo software (BD Biosciences, v10.8).

### RNA sequencing and analysis of sorted lung cells

Mice lungs were harvested, and a piece of the lung tissue was kept for baseline measurement of overall gene expression in the lung. The rest of the lung was dissociated and sorted into four distinct populations (CD45^+^ for leukocytes, CD45^–^CD31^+^CD326^–^ for endothelial cells, CD45^–^CD31^–^CD326^+^ for epithelial cells, and CD45^–^CD31^–^CD326^–^CD140a^+^ for fibroblasts) as described above. Total RNA was extracted using standard TRI Reagent (MilliporeSigma) RNA extraction protocol. The portion of lung tissue was fully disrupted prior to RNA extraction using the TissueLyser II (Qiagen, Germantown, MD, USA). RNA quality was verified in-house using the Fragment Analyzer System (Agilent) before submitting to GENEWIZ/Azenta (South Plainfield, NJ, USA) for library preparation and sequencing. Briefly, RNA quality was further validated with TapeStation (Agilent). Libraries were prepared with poly-A selection, and strand-specific RNA sequencing libraries were built using the NEBNext Ultra II Directional RNA Library Prep Kit (New England Biolabs, Ipswich, MA, USA) and sequenced on HiSeq 4000 sequencer (Illumina, San Diego, CA, USA) producing 2 × 150 bp paired-end reads.

Raw fastq files were aligned to an ensembl cDNA reference transcriptome (release-105) with kallisto (-b 30) (43). Compressed transcript tables output from kallisto were then aggregated and normalized for gene-level quantification and differential expression analysis as described by Pimentel *et al.* (44). To generate sample correlation heatmaps, we first calculated a z-score for each gene's expression in each sample's distribution of quantified genes using the native R function, ‘scale’. Next, these z-scores were used by the R ‘cor’ function to calculate pairwise sample correlations. Finally, correlation values were visualized using ‘corrplot’ (Github: taiyun/corrplot).

### Single-cell RNA sequencing

Mouse lung was collected and stored into MACS Tissue Storage Solution on ice while collecting lungs from multiple mice. An enzymatic dissociation solution was prepared with 100 μl of Enzyme D, 15 μl of Enzyme A (Miltenyi Biotec, Lung Dissociation Kit), 250 U ml^–1^ Collagenase IV, and 2.3 ml of DMEM. Collected mouse lung was dissociated to single-cell suspension using gentleMACS C Tubes and a gentleMACS Octo Dissociator with the enzymatic dissociation solution under the gentleMACS program 37C_m_LDK_1. Dissociated cells were filtered through a 70-μm strainer and centrifuged at 500 × g at 4°C for 10 min. After discarding the supernatant, RBCs were lysed using RBC lysis buffer, followed by washing with DMEM (500 × g, 4°C, 10 min). Next, the cell pellet was suspended in a flow cytometry buffer. Live singlet cells were collected using a BD FACSAria Fusion flow cytometer from enzymatically dissociated lung cells stained with 30 nM SYTOX Green (Thermo Fisher Scientific). A scRNA-seq library was prepared with Chromium Single Cell 3′ Reagent Kits V2 (10X Genomics, Pleasanton, CA, USA) according to the manufacturer's protocol. One thousand cells for each group (PBS and ASO-treated) were targeted during library preparation. The prepared library was sequenced on a HiSeq 4000 sequencer.

Raw fastq reads were processed using the DolphinNext Single Cell-10X Genomics Pipeline (45). Specifically, read1 was used to extract cell barcode and unique molecule identifier (UMI) information. Read2 which contains cDNA information was mapped to GRCm38 (46) with Gencode M25 (GRCm38.p6) annotation (46) using STAR (47). On average 75.5% of the reads were uniquely mapped. Cell barcodes with more than 3,000 uniquely mapped reads were kept in the downstream analysis. Gene expressions were quantified and UMI deduplicated using End Sequence Analysis Toolkit (ESAT, github/garber-lab/ESAT) (48).

The final cell by gene matrix was analyzed using Seurat package in R environment (49,50). Cell barcodes with more than 1,000 UMIs, more than 200 and less than 2,500 unique genes, and less than 5% mitochondrial gene content were deemed as good quality cells. The final dataset contains 867 cells from the sample treated with PBS and 970 cells treated with Malat1 LNA gapmer. Gene expressions were normalized by natural-log transformation. Cells were then dimensionally reduced with Principal Component Analysis (PCA) using the top 2,000 highly variable genes identified by the vst method in Seurat, and embedded with UMAP (51) using the first 15 principal components. Clusters were determined with K-Nearest Neighbor (KNN) graph.

Differential gene expression (DE) analysis was performed using edgeR (52) comparing Malat1 LNA gapmer treated cells with PBS treated cells per cell type. DE analysis was only performed on genes that were expressed in at least 10% of the cells in each cell type.

### RNA isolation and RT-qPCR assay

Tissues were homogenized in 1 ml of TRI Reagent with 5-mm steel balls for 2 × 2 min at 30 Hz using TissueLyser II. Samples were incubated for 10 min at room temperature, followed by adding 100 μl 1-bromo-3-chloropropane (MilliporeSigma). Samples were thoroughly mixed by vortexing, followed by a 20 min incubation at room temperature. After incubation, samples were centrifuged at 14 000 × g at 4°C for 15 min, the upper aqueous phase was transferred to a new tube, and 1 volume of isopropanol (MilliporeSigma) was added. Samples were then incubated for 30 min at room temperature, followed by centrifugation at 16 000 × g at 4°C for 30 min. After centrifugation, the supernatant was discarded, and the pellet was washed twice with 1 ml of cold 75% ethanol (MilliporeSigma). The pellet was air-dried for 20 min, resuspended in nuclease-free water, and quantified on a Nanodrop (Thermo Fisher Scientific). 500 ng of RNA was used for cDNA generation with a High-Capacity cDNA Reverse Transcription Kit (Thermo Fisher Scientific) according to the manufacturer's protocol. qPCR reactions were performed using iTaq Super Mix (BioRad, Hercules, CA, USA) on the CFX96 Real-Time Systems (BioRad) under the following condition: initial denaturation at 95°C for 3 min, followed by 40 cycles of denaturation at 95°C for 10 s, and annealing/extension at 60°C for 30 s. RT-qPCR primers and probes used in this study were purchased from the Integrated DNA Technologies (IDT, Coralville, Iowa, USA) as follows: *Ppib* (Mm.PT.58.29807961); *Malat1* (Mm.PT.58.10540953.g); *Cd47* (forward 5′-AAATGGATAAGCGCGATGCC-3′, reverse 5′-TGAAGGCCGTGCGGTTT-3′); *Cd98* (forward 5′-GGCTGAGTGGCAGAATATCA-3′, reverse 5′-GTCGCTGGTGGATTCAAGTA-3′); *Mfap4* (forward 5′-ACCTTGGCCTCATCACTTTAC-3′, reverse 5′-CAGTAGCCGTGGTGAGTATTG-3′); *Adam33* (Mm.PT.58.5867405); *Il6* (Mm.PT.58.5867405). The raw Ct values of RT-qPCR experiments are shown in Supplementary Table S2.

### Quantification of LNA gapmers from mouse tissues

Tissues were homogenized in 30 μl mg^–1^ radioimmunoprecipitation assay buffer (Alfa Aesar, Haverhill, MA, USA) with 5-mm steel balls for 2 × 2 min at 30 Hz using TissueLyser II. Samples were incubated for 1 h on ice and vortexed every 10 min, then subsequently centrifuged at 16 000 × g at 4°C for 30 min. The supernatant was transferred into a new tube and stored at −80°C until subsequent quantification analysis.

The LNA gapmer concentrations in the tissue were quantified with the SplintR qPCR assay following our previous work (53). Ligation probe and qPCR primers and probe were purchased from the IDT, and sequences are as follows: Malat1 Ligation probe A (5′-CTCGACCTCTCTATGGGCAGTCACGACGCATTCAG-3′); Malat1 Ligation probe B (5′-pTGAACTAGCTGAGTCGGAGACACGCAGGGCTTAA-3′); Malat1 SplintR qPCR probe set (forward 5′-GCTCGACCTCTCTATGGGC-3′, reverse 5′-TTAAGCCCTGCGTGTCTCC-3′, double-quenched probe 5′-/FAM/TCACGACGC/ZEN/ATTCAGTGAACTAGCTGAGTC/IABkFQ/-3′). p is a 5′ phosphate group, FAM is 6-carboxyfluorescein, ZEN is an internal quencher, and IABkFQ is Iowa Black quencher.

### Statistical analyses

Arithmetic mean (Mean) and standard deviation (SD) were used for RT-qPCR assay and quantification of LNA gapmers; geometric mean (Geo mean) and SD were used for flow cytometry analysis. For comparison of two groups, an unpaired *t*-test was performed using a Two-stage linear step-up procedure of Benjamini, Krieger, and Yekutieli. A one-way ANOVA was performed with a Dunnett's multiple comparison test to compare more than two groups. Graph generations and statistical tests were performed in GraphPad Prism V 9.3.0 (GraphPad Software, San Diego, CA, USA).

## RESULTS

### Intratracheally administered gapmer ASOs induce silencing in mouse lung tissue

**Figure 1.**
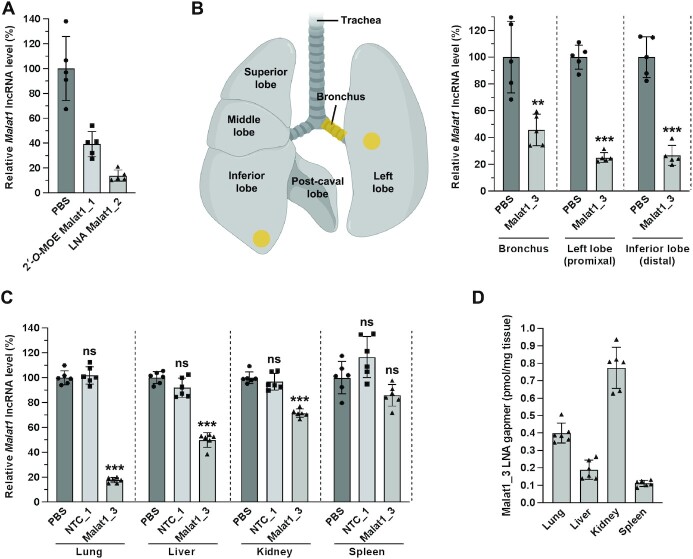
Silencing efficacy in the lung and other tissues after intratracheal administration of gapmer ASOs targeting *Malat1*. (**A, B**) Mice were intratracheally administered 7.5 nmol of 2′-*O*-MOE ASO (∼2.2 mg kg^–1^), LNA ASO (∼1.5 mg kg^–1^), or PBS (*n* = 5), and lungs were collected 2 days (A) or 6 days (B) later. The silencing of *Malat1* lncRNA was measured with RT-qPCR and normalized to *Ppib* mRNA level. Data are presented relative to the PBS group. (**B**) The uniformity of silencing in different regions of the lung was analyzed by collecting punches from lung regions (highlighted in yellow) before measuring RNA levels. (**C, D**) Mice were intratracheally administered 10 nmol (∼2.1 mg kg^–1^) of ASO or PBS (*n* = 5), and tissues were collected 1 week later. (**C**) The silencing of *Malat1* lncRNA was measured with RT-qPCR in the lung, liver, kidney, and spleen and normalized to *Ppib* mRNA level. Data are presented relative to the PBS group in each tissue. (**D**) The concentration of Malat1 ASO in the lung, liver, kidney, and spleen was quantified by the SplintR qPCR assay. Data are presented as arithmetic mean (Mean) ± standard deviation (SD) with values of the individual animals as dots. **P < 0.01, ***P < 0.001 versus the PBS group (unpaired *t*-test in panel B, one-way ANOVA in panel C). 2′-*O*-MOE, 2′-*O*-methoxyethyl; LNA, locked nucleic acid; PBS, phosphate-buffered saline; NTC, non-target control. Illustration was created with BioRender.com.

We tested the silencing ability of 2′-*O*-MOE and LNA-modified gapmer ASOs after intratracheal administration to mouse lung. Gapmer ASO sequences targeting *Malat1* were adapted from previous research (54). A 7.5-nmol dose of 2′-*O*-MOE gapmer targeting the long noncoding RNA (lncRNA) *Malat1* showed 60% silencing by 2 days after administration, while the same dose of a corresponding LNA gapmer showed 90% silencing (Figure 1A). We, therefore, focused the remainder of our work on LNA-modified ASOs.

Silencing by LNA gapmer in the different regions of lung tissue was measured. 7.5 nmol of LNA gapmer targeting *Malat1* delivered to mouse lung by intratracheal instillation. After 6 days, tissues from the bronchus, proximal region of the left lobe, and distal region of the inferior lobe were collected in the lung, and analyzed with RT-qPCR. Similar silencing was observed both in proximal and distal regions of the lung (75%), and relatively lower silencing was observed in the bronchus (55%) (Figure 1B).

In order to test the systemic silencing efficacy of locally delivered LNA gapmer in the lung, we delivered 10 nmol of LNA gapmer targeting *Malat1* by intratracheal instillation to 8-week-old female FVB mice. After 1 week, mice were euthanized, and tissues from the lung, liver, kidney, and spleen were analyzed with RT-qPCR. The highest silencing of the target gene *Malat1* lncRNA was observed in the lung, the locally administered site (Figure 1C). The systemic effect was also examined in the liver, kidney, and spleen. Previous research reported systemic exposure to LNA ASO and high silencing in liver after local administration to the lung (55) – however, in contrast to that work, we see robust silencing in the lung (>80%), compared with only moderate silencing in the liver (50%) and <30% silencing in kidney and spleen (Figure 1C). After systemic administration of non-conjugated ASOs by intravenous or subcutaneous injection, silencing is typically most robust in the liver (56). However, as shown here, local administration to the lung provides an opportunity to achieve excellent silencing with relatively low systemic exposure. Therefore, local administration to the lungs may reduce the risk of adverse effects following systemic administration and improve overall therapeutic index.

To determine whether any correlation exists between ASO uptake and silencing efficacy, ASO levels were quantitated in each tissue using a SplintR qPCR assay (53) after intratracheal administration. LNA gapmer was present at the highest concentration in the kidney, followed by the lung, liver, and spleen in order of decreasing concentration among the analyzed tissues (Figure 1D). There was no correlation between silencing efficacy and tissue concentration: The lung showed substantially higher silencing than the kidney, despite higher ASO concentration in the kidney. We speculate that this discrepancy between silencing efficacy and concentration may be driven by reasons including timing (the concentration was measured 1 week after administration, so it may not reflect the total area under the curve in terms of tissue exposure) or simply because gross tissue concentration might correlate poorly with functional uptake leading to silencing efficacy, *i.e*. although the kidney has higher gross uptake, the ASOs may be sequestered extracellularly or in a subcellular compartment that does not allow them to be active, as previously suggested (57,58).

In the same way that different tissues are differentially susceptible to ASO-induced gene silencing, different cell types within each of these complex tissues may respond differently to ASO administration. The ability to produce robust effects in specific cell populations is of central importance to therapeutic interventions, and yet this aspect is poorly understood in the lung. We, therefore, set out to study cell-type-specific silencing of ASOs in the lung using several independent methods.

### Flow cytometry analysis of mouse lung reveals fibroblasts are the most susceptible lung cell type for ASO-induced gene silencing

**Figure 2.**
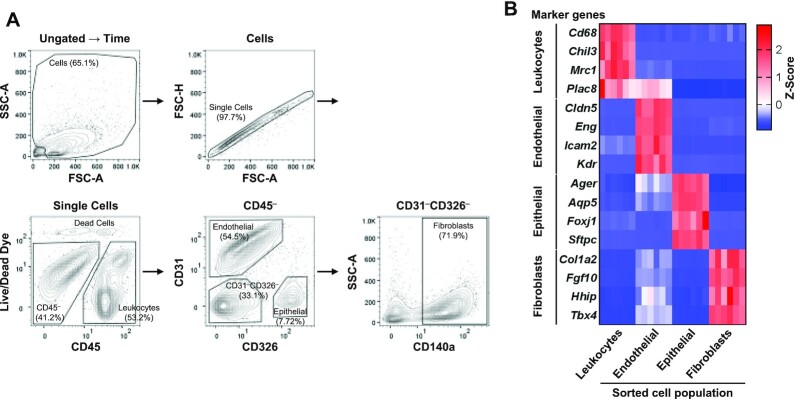
Flow cytometry analysis strategy to identify leukocytes, endothelial cells, epithelial cells, and fibroblasts of mouse lung. Enzymatically dissociated mouse lungs were stained with VioGreen-CD45, APC-CD31, FITC-CD326, and PE-Vio770-CD140a antibodies, and SYTOX Blue Live/Dead cell dye. (**A**) After time gating, debris and non-single cells were excluded by gating on FSC-A × SSC-A and FSC-A × FSC-H. The cell population of different cell types was separated as follows: leukocytes (CD45^+^), endothelial cells (CD45^–^CD31^+^CD326^–^), epithelial cells (CD45^–^CD31^–^CD326^+^), and fibroblasts (CD45^–^CD31^–^CD326^–^CD140a^+^). A ratio of each cell population to the parent population was presented. (**B**) Each cell population was collected using the fluorescence-activated cell sorter, and RNA was isolated. RNA sequencing analysis revealed the enriched gene expression of cell-type-specific markers in each cell type. FSC, forward scatter; SSC, side scatter; A, area; H, height.

To further investigate lung silencing, we first established a flow cytometry assay to enable efficient exploration of cell-type-specific silencing in the lung. Analysis of leukocytes, endothelial cells, and epithelial cells in the mouse lung was previously reported, however, fibroblast populations were not analyzed (59). In previous studies, platelet-derived growth factor receptor alpha (PDGFRα, CD140a) was used as a fibroblast lineage marker combined with other cellular markers in the lung and colon (59–61). Therefore, we separated and analyzed fibroblasts using the CD140a antibody among the CD45^–^CD31^–^CD326^–^ cell population. Figure 2A shows the gating strategy for each lung cell population of leukocytes, endothelial cells, epithelial cells, and fibroblasts. To verify that our gating strategy resulted in the expected cell populations, we analyzed each sorted cell population using RNA-seq. As expected, cell-type-specific markers were enriched in each sorted cell population. For example, the CD45-positive cells were enriched for additional leukocyte markers including *Cd68*, *Chil3*, *Mrc1* and *Plac8*. CD31-positive cells were enriched for additional endothelial cell markers including *Cldn5*, *Eng*, *Icam2* and *Kdr*. CD326-positive cells were enriched for additional epithelial cell markers including *Ager*, *Aqp5*, *Foxj1* and *Sftpc*. Gratifyingly, we observed that CD140a-positive cell populations were enriched for additional fibroblast markers including *Col1a2*, *Fgf10*, *Hhip* and *Tbx4* (Figure 2B) (18,29,62–67). Additional details of the RNA-seq from cell populations are given in Supplementary Figure S1. In all, our flow cytometry protocol provides rapid access to meaningful cell-type-specific populations from the lung.

Although ASO-mediated silencing of target genes in each cell type can be measured by RT-qPCR after flow cytometric sorting and collection of cell populations, the throughput of this process is relatively low and requires intensive work. We, therefore, designed a strategy to measure silencing efficacy without the need to collect cells, by detecting the protein level of the target gene in the flow cytometer directly. Since *Malat1* lncRNA does not encode a protein, another model gene was required for the flow cytometry approach. We selected *Cd47* as an appropriate model gene since it has a number of valuable characteristics for flow cytometry analysis: (i) it is ubiquitously expressed in most cell types in the lung (Supplementary Figure S2A), (ii) it is localized at the cell plasma membrane, (iii) mice have no severe deficit when *Cd47* is silenced in a normal context, (iv) CD47 antibodies are readily available (68,69).

We screened ASOs to find potent inhibitors of *Cd47* expression. Gapmer ASO sequences for the target gene were designed using the LNCASO online tool (https://iomics.ugent.be/pjdev/) and selected by transfection into cultured mouse embryonic fibroblast cells. After screening 24 sequences, we narrowed our search to four lead compounds; these four were tested in mice and enabled us to identify a lead compound (Supplementary Figure S3A).

**Figure 3.**
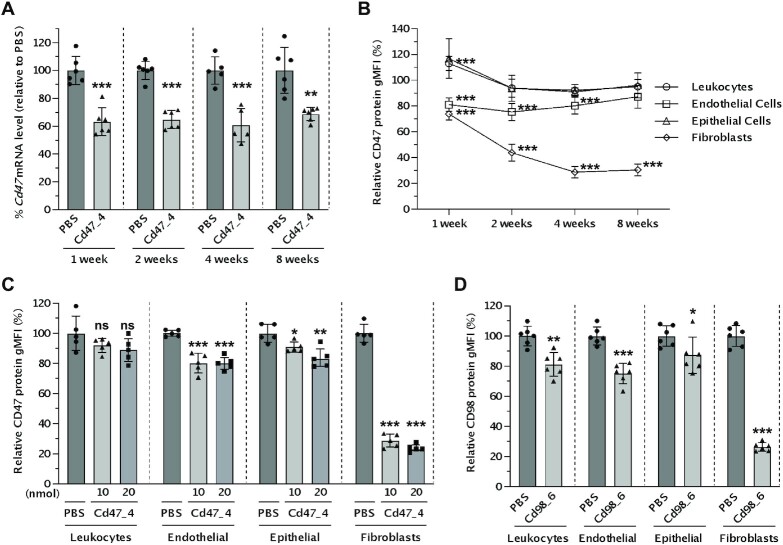
Silencing efficacy in different cell types of mouse lung after intratracheal administration of LNA gapmer ASOs. (**A**−**C**) Mice were intratracheally administered 10 nmol (∼2.1 mg kg^–1^) (A, B) or indicated dose (∼2.1 or 4.3 mg kg^–1^) (C) of ASO targeting *Cd47* or PBS (*n* = 6 for 1, 2, 8 weeks, *n* = 5 for 4 weeks), and lungs were collected after indicated time point (A, B) or 4 weeks (C). (**A**) RNA was isolated from bulk lung. The silencing of *Cd47* mRNA was measured with RT-qPCR and normalized with *Ppib* mRNA level. Data are presented relative to the PBS group at each time point. (**B, C**) Single cells isolated from mice lungs were stained with VioGreen-CD45, APC-CD31, FITC-CD326, PE-Vio770-CD140a, and PE-CD47 antibodies, and SYTOX Blue Live/Dead cell dye, and then analyzed by flow cytometry. The CD47 protein level of ASO-treated groups is presented relative to the PBS group of each time point by cell type. (**D**) Mice were intratracheally administered 20 nmol (∼4.3 mg kg^–1^) of ASO targeting *Cd98* or PBS (*n* = 6), and lungs were collected after 4 weeks subsequently analyzed by flow cytometry staining with VioGreen-CD45, APC-CD31, FITC-CD326, PE-Vio770-CD140a, and PE-CD98 antibodies, and SYTOX Blue Live/Dead cell dye. The CD98 protein level of ASO-treated groups is presented relative to the PBS group of each cell type. Data are presented as Mean ± SD (A) or geometric mean (Geo mean) ± SD (B–D) with values of the individual animals as dots. *P < 0.05, **P < 0.01, ***P < 0.001 versus the PBS group (unpaired *t*-test for A, B, D; one-way ANOVA for C). gMFI, geometric mean fluorescence intensity.

The mice were intratracheally administered a single dose of 10 nmol of LNA gapmer targeting *Cd47*. After 1, 2, 4 and 8 weeks, mice were euthanized, and the lungs were collected and analyzed by RT-qPCR and flow cytometry (Figure 3A and B). Overall, the silencing effect at the level of *Cd47* mRNA by our lead LNA gapmer was moderate (40%) when observed in bulk lung tissue from 1 week to 8 weeks after administration (Figure 3A). We further investigated the silencing of CD47 protein within specific cell populations in the lungs by flow cytometry. To do this, we harvested lungs and immediately prepared a single-cell suspension using an enzymatic dissociation solution with gentle mechanical disruption, then labeled these cells with fluorescent antibodies for cell-type-specific markers and target protein (CD45, CD31, CD326, CD140a, and CD47, see Materials and Methods). Flow cytometry revealed that fibroblasts showed the highest silencing (70%) among lung cell types (Figure 3B). Remarkably, the silencing of CD47 protein levels in fibroblasts was maintained for up to 8 weeks after a single dose of ASO.

A single dose of 20 nmol of LNA gapmer targeting *Cd47* was compared to 10 nmol at 4 weeks. A trend of enhanced silencing was observed in epithelial cells and fibroblasts, but the difference was slight (Figure 3C).

To ensure that this effect was not an artifact of CD47 biology, we repeated the study using an additional broadly expressed cell-surface marker. We tested the consistency of expression of cell surface proteins CD29 and CD98 and selected CD98 as a good candidate since it is ubiquitously expressed in lung cell types (Supplementary Figure S2B and C) (70). We, therefore, screened ASOs to find potent inhibitors of *Cd98* expression. From 24 sequences, we narrowed our search to six lead compounds; these six were tested in mice and enabled us to identify a lead compound (Supplementary Figure S3B).

To explore cell-type-specific silencing of CD98, we carried out intratracheal administration of 20 nmol LNA gapmer targeting *Cd98* to mice. After 4 weeks, lung cells were analyzed by flow cytometry. The ASO targeting *Cd98* showed efficient silencing of CD98 protein in lung fibroblasts (Figure 3D). These results strongly suggest that CD140a-positive fibroblasts are favourable cell targets for ASO-mediated gene silencing.

### Single-cell RNA sequencing reveals robust silencing in mouse lung fibroblasts

For further verification, we employed another approach, scRNA-seq. scRNA-seq is a powerful approach to explore the biology of individual cells in the context of complex tissues. Since the lung has one of the most heterogeneous cell contexts, we decided to take advantage of scRNA-seq as an independent method to study the cell-type-specific effects of ASOs.

For measuring gene silencing in a scRNA-seq approach, it is good to target abundant transcripts such as *Malat1* since the sequencing depth in scRNA-seq is low compared to normal RNA-seq analysis – we obtained an average of ∼130 000 reads/cell, which means that only highly expressed transcripts will be represented to a level that allows meaningful measurements of gene silencing.

**Figure 4.**
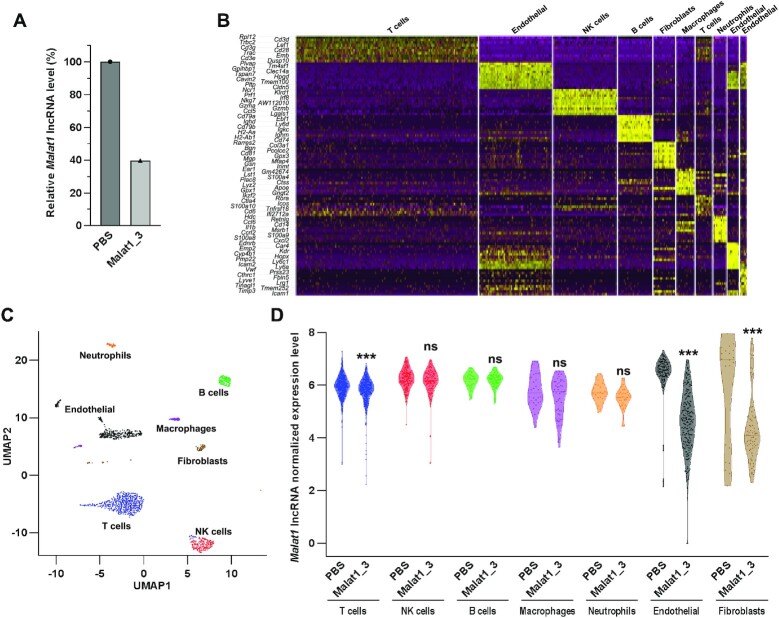
scRNA-seq analysis of mouse lung after intratracheal administration of LNA gapmer ASO targeting *Malat1*. Mice were intratracheally administered 7.5 nmol (∼1.6 mg kg^–1^) of ASO or PBS (*n* = 1), and lungs were collected after 2 days. A small piece of mouse lung (30 mg) was collected for RNA isolation, and single cells were isolated from the rest of the lung using flow cytometric sorting. Isolated cells were subjected to scRNA-seq analysis (867 cells in PBS and 970 cells in the ASO-treated group). (**A**) Silencing of *Malat1* lncRNA in bulk lung tissue was measured by RT-qPCR and normalized with *Ppib* mRNA expression. Data are presented relative to the PBS group. (**B**) Heatmap of differentially expressed genes between each clustered cell type (both treatment groups combined). (**C**) Annotation of algorithmically identified lung cell clusters (both treatment groups combined). (**D**) The normalized expression level of *Malat1* lncRNA in each cell type. Data are presented as violin plot with the median as mid-line and values of the individual cell as dots. ***False discovery rate (FDR)-adjusted *P* < 0.001 versus the PBS group.

We administered an LNA gapmer ASO targeting *Malat1* to mice. Since *Malat1* is relatively easy to silence, we used a low dose of ASO (7.5 nmol) in order to prevent saturation of silencing among different cell types. After 2 days, mouse lungs were collected and analyzed. Approximately 60% silencing of *Malat1* lncRNA was achieved in bulk lung tissue (Figure 4A). We gently dissociated cells as described above, isolated live singlet cells using a flow cytometer, and prepared a scRNA-seq library using an emulsion-based protocol (see Materials and Methods) (71).

Cells clustered by their gene expression profiles, and each cluster was annotated by marker gene expression (Figure 4B and C). We compared the extent of gene silencing in each cell population. We observed high ASO-mediated gene silencing in fibroblasts and endothelial cells. This is consistent with our observations from flow cytometry. We observed negligible silencing in T cells, NK cells, B cells, macrophages, and neutrophils under these conditions (Figure 4D). Epithelia were not observed in this single-cell experiment as the enzymatic dissociation solution used did not sufficiently dissociate the epithelial cells from the lungs.

Thus, across two independent analytical approaches (flow cytometry and single-cell RNA sequencing), we observed robust and durable gene silencing in lung fibroblasts after intratracheal administration of LNA gapmer ASOs targeting *Cd47, Cd98* and *Malat1*. In addition, endothelial cells of the lung also showed substantial gene silencing.

### Robust silencing of fibroblast-specific genes measured in bulk lung tissue

As another method of demonstrating robust silencing in fibroblasts, we hypothesized that targeting a gene selectively expressed in fibroblasts would be readily detectable even in bulk lung tissue. By contrast, if a gene is widely expressed in most or all lung cell types but silencing operates in only a subset of cells, this would result in apparently poor overall silencing when measured in bulk lung tissue.

**Figure 5.**
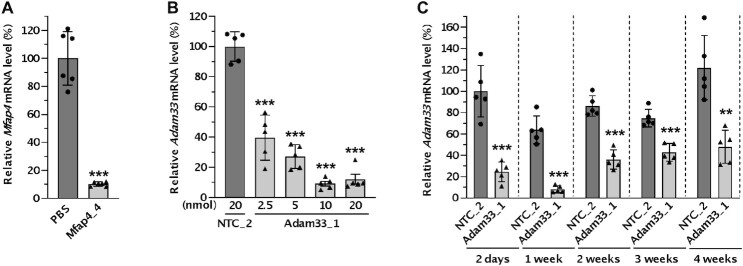
Verification of robust silencing in lung fibroblasts by intratracheally administered LNA gapmer ASOs targeting multiple genes. (**A**) Mice were intratracheally administered 20 nmol (∼4.3 mg kg^–1^) of ASO targeting *Mfap4* or PBS (*n* = 6), and lungs were collected after 1 week. The silencing of *Mfap4* mRNA was measured with RT-qPCR and normalized with *Ppib* mRNA level. Data are presented relative to the PBS group. (**B, C**) Mice were intratracheally administered indicated (∼0.5–4.0 mg kg^–1^) (B) or 10 nmol (∼2.0 mg kg^–1^) (C) of ASO targeting *Adam33* or NTC (n = 5), and lungs were collected after 1 week (B) or the indicated time point (C). The silencing of *Adam33* mRNA was measured with RT-qPCR and normalized with *Ppib* mRNA level. Data are presented relative to the NTC group at each time point (B) or the NTC at the 2-day time point (C). Data are presented as Mean ± SD with values of the individual animals as dots. **P < 0.01, ***P < 0.001 versus the PBS or NTC group (unpaired *t*-test for A, C, one-way ANOVA for B).

To test this hypothesis, we first selected *Mfap4*, which exhibits enriched expression in fibroblasts as shown by the LungMap database (38). 1 week after intratracheal administration of 20 nmol of LNA gapmer targeting *Mfap4*, robust silencing (90%) was observed even in bulk lung tissues measured by RT-qPCR (Figure 5A and Supplementary Figure S3C).

*Adam33* is another target gene expressed in lung fibroblasts (72); a soluble form of this metalloprotease has been shown to induce asthma-related airway remodeling (73). Mice were intratracheally administered a single dose of 2.5–20 nmol of LNA gapmer targeting *Adam33* (41). After 1 week, the silencing efficacy of *Adam33* was analyzed by RT-qPCR in bulk lung tissues. The LNA gapmer showed silencing of the target gene *Adam33* dose-dependently until 10 nmol, but doses higher than 10 nmol did not increase silencing efficacy (Figure 5B). We selected the 10 nmol dose and carried out a timecourse experiment to examine the duration of the effect. The LNA gapmer showed robust silencing of the target gene *Adam33*, which peaked at 1 week (>90%) and lasted at least 4 weeks (Figure 5C).

## DISCUSSION

In this study, we used independent approaches to evaluate the efficacy of ASOs in distinct lung cell types—using flow cytometry (Figures 2 and 3) and scRNA-seq (Figure 4). These approaches offer a way to study oligonucleotide therapeutics in the different cell types that make up the lung, and may be applicable to other tissues. According to recent scRNA-seq databases, CD47 and CD98, the model genes used in this study, are expressed ubiquitously in most tissues and cell types in humans and mice (74,75). Moreover, the knockout (KO) mouse model is available, which can be helpful for establishing and validating assay conditions.

The flow cytometry approach allows a more refined analysis of the effects of oligonucleotide therapeutics at the cell population level instead of entire tissues. This experiment based on silencing of membrane-expressed marker genes, provides higher throughput and requires less effort than FACS-based collection of cell populations followed by subsequent analysis by RT-qPCR or other approaches. Using different antibody panels of interest could extend the method further, provide a method to explore additional cell types and more defined subtypes.

In addition, to the best of our knowledge, examination of gene silencing by ASOs using scRNA-seq has not been previously described. This approach can allow us to study the action of oligonucleotide therapeutics in previously unanalysable cell types that escape conventional analyses. For example, scRNA-seq analysis of human blood cells found and validated six dendritic cell subtypes and four monocyte subtypes, including new dendritic cell subsets and additional monocyte populations previously uncharacterized (76). Moreover, using scRNA-seq after treatment with oligonucleotide therapeutics allows simultaneous measurement of a gene directly modulated by oligonucleotide therapeutics and downstream genes affected by target-gene silencing on a transcriptome-wide level in each cell.

Understanding how antisense oligonucleotides behave in each cell type in different tissues through appropriate analytical methods will promote the development of novel oligonucleotide therapeutics where the approach is well aligned with the cells showing the highest efficacy. By scrutinizing specific cell populations, we demonstrated that LNA gapmer ASOs elicit robust silencing in lung fibroblasts. Similar to the lungs, most organs are made up of many different cell types, with distinct preferences for ASO uptake. For example, unconjugated PS-modified ASOs show preferential uptake in Kupffer, endothelial, and stellate cells in the liver (77), while GalNAc-conjugated ASOs show preferential uptake in hepatocytes (78). The cell-type-specific effects of ASOs have also been explored in the brain, another complex tissue made up of diverse cell types—Jafar-Nejad *et al.* showed that centrally administered ASO produced high silencing in neurons, oligodendrocytes, microglia, and astrocytes at the cell-type-specific level using an in-situ hybridization approach and RT-qPCR after magnetic cell isolation (79). Interestingly, the cell-type-specific distribution and activity of ASOs were overall well maintained across the three species used – mouse, rat, and non-human primate (79). However, methods that rely on cell fractionation or microscopic assays have limited quantification capability and take intensive effort. Therefore, applying the approach introduced in this study—flow cytometry and scRNA-seq—might reveal more precise and cost-effective information about behaviors of ASO therapeutics at a single-cell level.

In recent years, many efforts have been made to improve the delivery of oligonucleotide therapeutics, including conjugation of functional ligands or antibodies, or lipid nanoparticle approaches (80). However, most assays relied on observation at the tissue level—increased accumulation in a specific organ and silencing in bulk tissue. Applying a cell-type-specific or single-cell approach to these efforts toward delivery enhancement could enable researchers to find approaches with significantly improved functionality at the cell-type-specific level even if the overall improvement is small at the bulk tissue level. An understanding of the cell types most susceptible to oligonucleotide targeting, in combination with knowledge of cellular contexts that contribute to disease states, will allow prioritization of indications most suitable for oligonucleotide therapeutic intervention.

The lung is an organ of interest for oligonucleotide therapeutics, but lung fibroblasts have not received much attention compared to epithelial or endothelial cells. Most preclinical and clinical research efforts have focused on epithelial cells. This is natural because many pulmonary diseases are associated with epithelial cells that cover a large surface area and perform gas exchange, the main function of the lungs. Encouraging observations have been reported but still need more improvement to target epithelial cells at a clinically meaningful level. For example, oropharyngeal administration of a cEt ASO targeting *Jag1* showed a significant reduction of *Jag1* mRNA (∼75% silencing) and downregulated the secretory club cell marker *Scgb1a1* in the lung, but this silencing in epithelial cells required multiple doses in a short period, three times 200 μg ASO in 1 week (18). Silencing efficacy within epithelial cells may see additional progress in the future, but in the meantime, an easier therapeutic strategy might be to focus on more susceptible cell types such as fibroblasts.

Fibroblasts in the lung have a major role in the pathogenesis of many lung diseases, including some forms of asthma and pulmonary fibrosis (34). We demonstrated robust and prolonged silencing of *Adam33*, a promising disease target in asthma which is expressed primarily in fibroblasts (Figure 5C). The potential of silencing disease-related target genes in the lung fibroblasts has already been proven in cell-type-specific KO mice models and through some additional oligonucleotide-based studies. For example, silencing of sphingosine kinase 1 only in fibroblasts using fibroblast-specific protein 1-Cre mice model showed that fibroblast-specific gene inhibition is sufficient to mitigate pulmonary fibrosis induced by bleomycin (81). A tamoxifen-induced deletion of the IL11 receptor in adult fibroblasts also showed reduced lung fibrosis and chronic inflammation in the bleomycin model (82). In addition, LNA gapmer ASOs targeting the lncRNA *DNM3OS* which is expressed in lung fibroblasts showed prophylactic and therapeutic effects on bleomycin-induced lung fibrosis (83).

Further research is required to uncover the mechanistic explanations behind the robust gene silencing observed in lung fibroblasts. Both cellular uptake and intracellular trafficking govern the productive uptake of ASOs (84). Investigations have revealed proteins that affect ASO activities in interstitial space, cell membrane, and intracellularly (6). However, more studies are needed to understand the cell-type-specific behaviours of ASOs. In addition to the LNA gapmer ASOs studied here, a systematic investigation of other scaffolds including fully modified siRNAs is also required. These efforts could help contribute to an understanding of the mechanisms as well as expand the application of oligonucleotide therapeutics.

Taken together, our results suggest that efforts to develop ASO therapeutics for the lung will maximize their likelihood of success by focusing on the modulation of gene expression in lung fibroblasts.

## DATA AVAILABILITY

The sequencing data in this paper are accessible through the Sequence Read Archive with the reference number PRJNA830496.

## Supplementary Material

gkac630_Supplemental_File
