# Supplementary material for: Intratracheally administered LNA gapmer antisense oligonucleotides induce robust gene silencing in mouse lung fibroblasts
Source: Nucleic Acids Res. 2022 Aug 3;50(15):8418–30. doi: 10.1093/nar/gkac630 (PMC9410908; doi:10.1093/nar/gkac630)
Supplement: gkac630_Supplemental_File [file gkac630_supplemental_file.pdf]

SUPPLEMENTARY DATA

Intratracheally administered LNA gapmer antisense oligonucleotides induce robust gene silencing in mouse lung fibroblasts

Minwook Shin[1], Io Long Chan[1], Yuming Cao[2], Alisha M. Gruntman[3,4,5], Jonathan Lee[1], Jacquelyn Sousa[1], Tomás C. Rodríguez[1], Dimas Echeverria[1], Gitali Devi[1], Alexandre J. Debacker[1], Michael P. Moazami[1], Pranathi Meda Krishnamurthy[1], Julia Rembetsy-Brown[1], Karen Kelly[1], Onur Yukselen[2], Elisa Donnard[2], Teagan J. Parsons[6,7], Anastasia Khvorova[1,6], Erik J. Sontheimer[1,6,8], René Maehr[6,7], Manuel Garber[2,6], and Jonathan K. Watts[1,8,9,*]

[1] RNA Therapeutics Institute, University of Massachusetts Chan Medical School, Worcester, MA, 01605, USA

[2] Program in Bioinformatics and Integrative Biology, University of Massachusetts Chan Medical School, Worcester, MA, 01605, USA

[3] Horae Gene Therapy Center, University of Massachusetts Chan Medical School, Worcester, MA, 01605, USA

[4] Department of Pediatrics, University of Massachusetts Chan Medical School, Worcester, MA, 01605, USA

[5] Department of Clinical Sciences, Cummings School of Veterinary Medicine at Tufts University, N. Grafton, MA, 01536, USA

[6] Program in Molecular Medicine, University of Massachusetts Chan Medical School, Worcester, MA, 01605, USA

[7] Diabetes Center of Excellence, University of Massachusetts Chan Medical School, Worcester, MA, 01605, USA

[8] Li Weibo Institute for Rare Diseases Research, University of Massachusetts Chan Medical School, Worcester, MA, 01605, USA

[9] Department of Biochemistry and Molecular Biotechnology, University of Massachusetts Chan Medical School, Worcester, MA, 01605, USA

* To whom correspondence should be addressed. Tel: +1-774-455-3784; Fax: +1-508-856-6696; Email: Jonathan.Watts@umassmed.edu

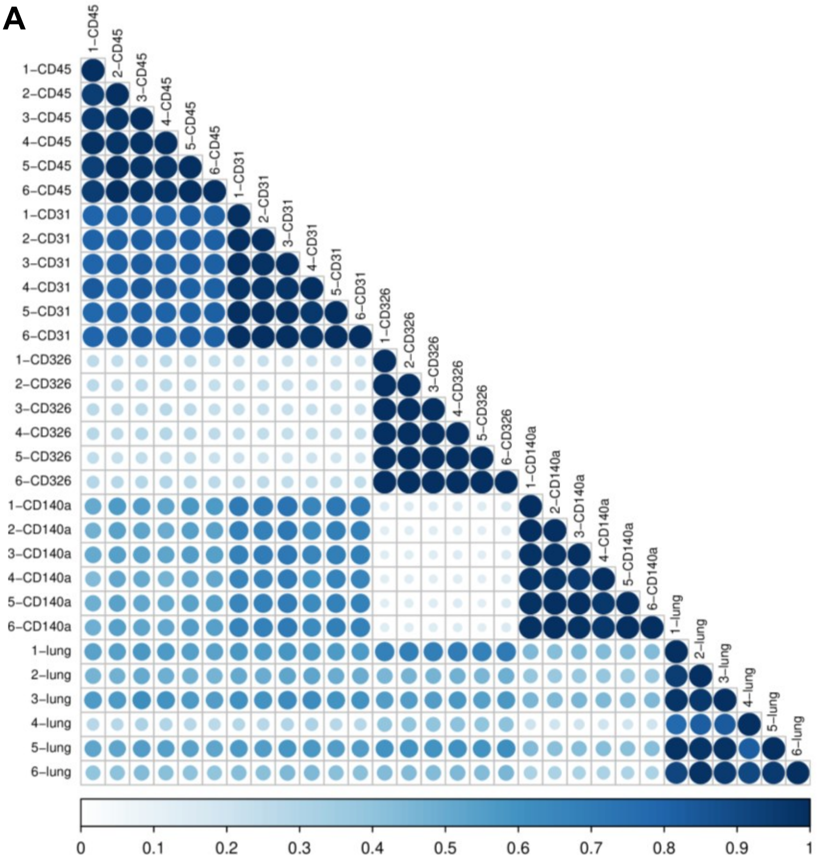

B

Comparison between marker$^+$ cell types	Genes co-expressed with marker
CD45$^+$ vs. other groups	2205
CD31$^+$ vs. other groups	1272
CD326$^+$ vs. other groups	2138
CD140a$^+$ vs. other groups	1861

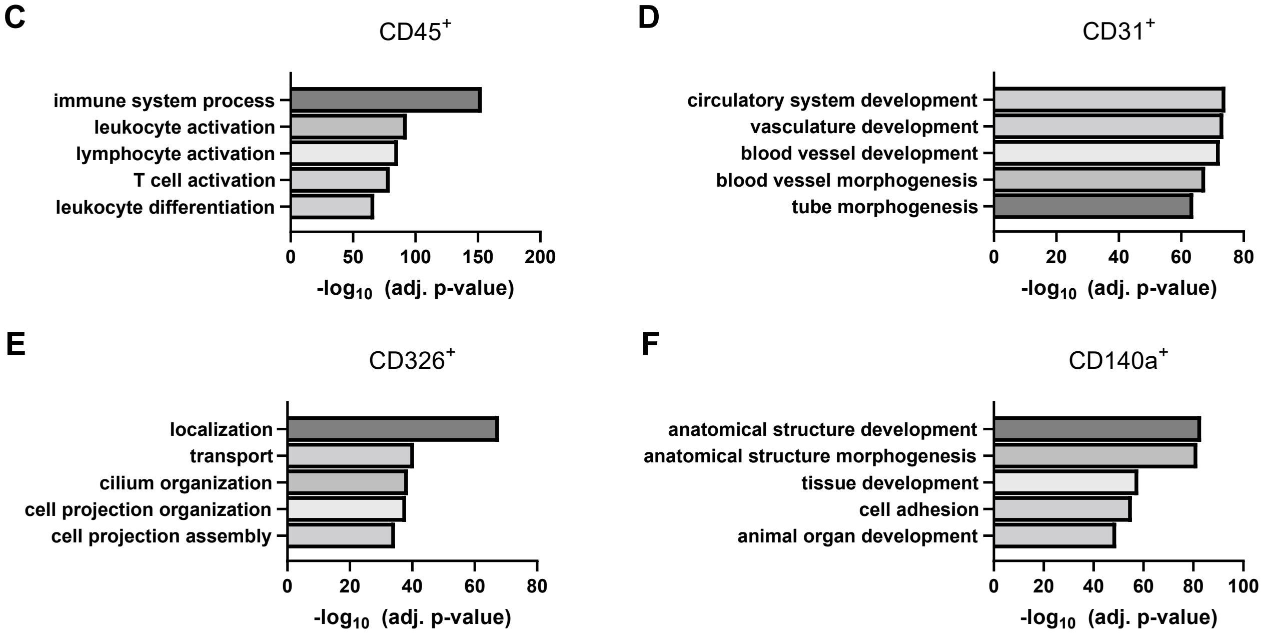

Supplementary Figure S1. Overview of RNA sequencing results. **(A)** Overall gene expression in each sorted cell-marker-positive group and bulk lung tissue correlates strongly with its own cell or tissue type between biological replicates. Scale represents Pearson correlation (0 to 1), and color intensity and size are proportional to correlation. Numbers 1-6 represent individual mice. **(B)** Differentially expressed genes that were upregulated in each group compared to all other groups (>2-fold, adjusted *P*-value<0.05). **(C-F)** Gene ontology analysis of the upregulated genes from panel B.

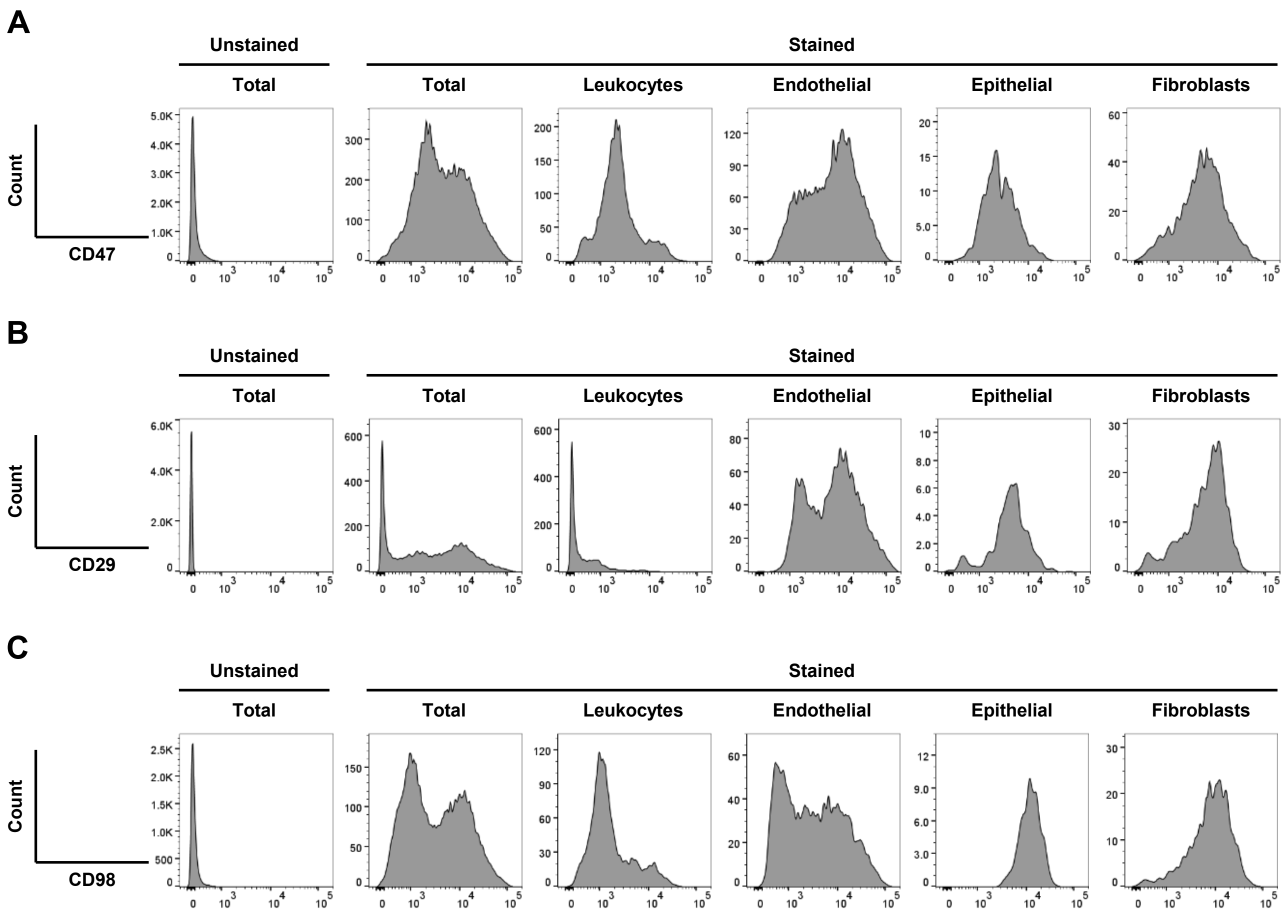

Supplementary Figure S2. Protein expression of model genes in each cell type. Enzymatically dissociated mice lungs were stained with VioGreen-CD45, APC-CD31, FITC-CD326, and PE-Vio770-CD140a antibodies, and SYTOX Blue Live/Dead cell dye along with an antibody for PE-CD47 **(A)**, PE-CD29 **(B)**, or PE-CD98 **(C)**. The protein expression translated from each model gene is presented in a histogram for each cell type. This data was used to select CD47 and CD98 as our model genes; CD29 was not considered ideal since a fraction of cells did not show positive staining.

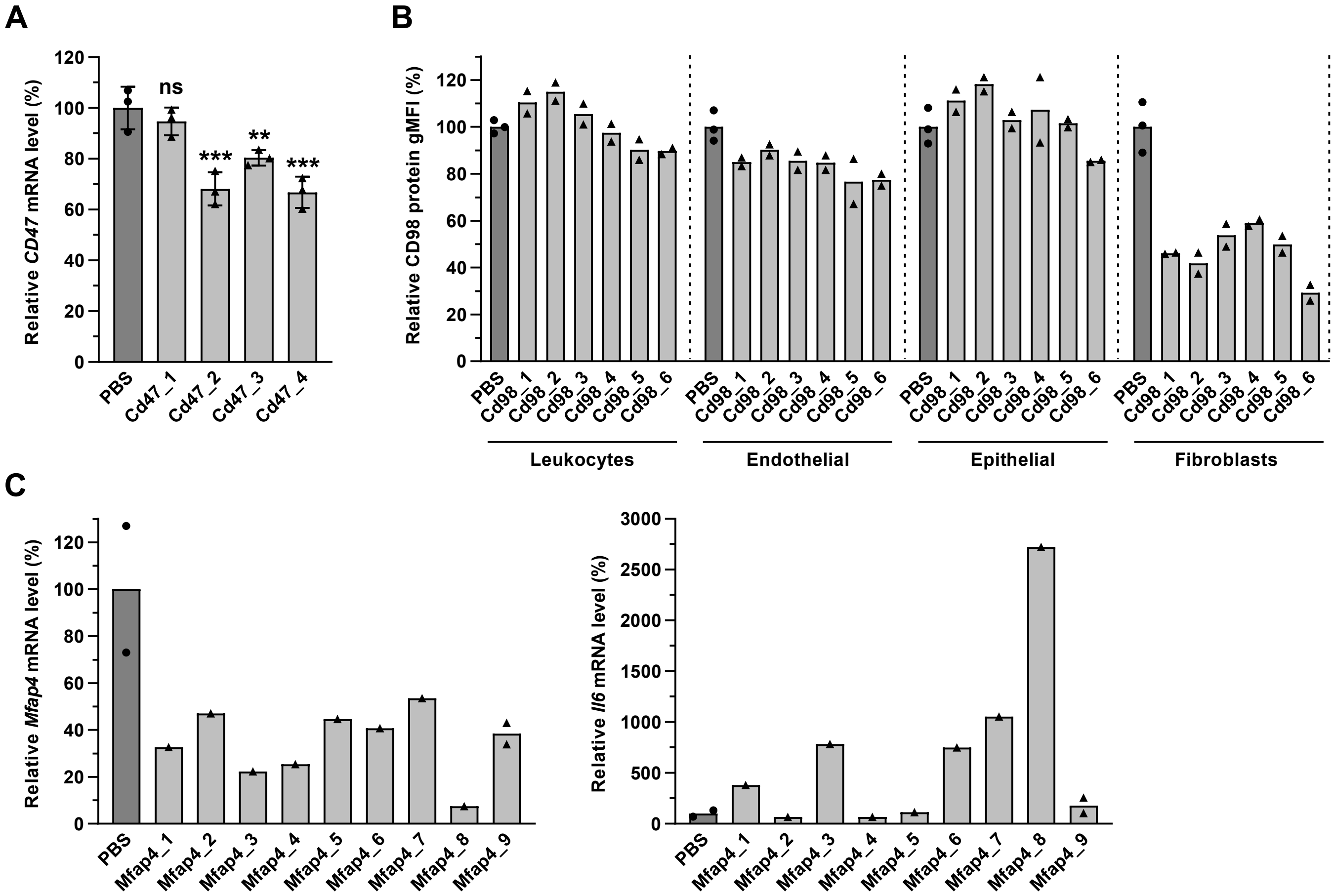

Supplementary Figure S3. In vivo screening for LNA gapmer ASOs to find lead compounds. **(A)** Mice were intratracheally administered 10 nmol (~2.2 mg kg^{-1}) of ASO targeting *Cd47* or PBS (n=3), and lungs were collected 2 days later**.** The silencing of *Cd47* mRNA was measured with RT-qPCR and normalized with *Ppib* mRNA level. Data are presented relative to the PBS group. **P <0.01, ***P <0.001 versus the PBS group (one-way ANOVA). **(B)** Mice were intratracheally administered 20 nmol (~4.3 mg kg^{-1}) of ASO targeting *Cd98* (n=2) or PBS (n=3), and lungs were collected after 4 weeks subsequently analyzed by flow cytometry staining with VioGreen-CD45, APC-CD31, FITC-CD326, PE-Vio770-CD140a, and PE-CD98 antibodies, and SYTOX Blue Live/Dead cell dye. The CD98 protein level of ASO-treated groups presented relative to the PBS group of each cell type. **(C)** Mice were intratracheally administered 20 nmol (~4.3 mg kg^{-1}) of ASO targeting *Mfap4* or PBS (n=1-2), and lungs were collected 2 days later. The silencing of *Mfap4* mRNA and induction of *Il6* mRNA were measured with RT-qPCR and normalized with *Ppib* mRNA level. Data are presented relative to the PBS group. Data are presented as Mean ± SD (A) or Geo Mean (B) or Mean (B) with values of the individual animals as dots.

Target	Oligo name	Sequence (5′-3′)	Calculated mass	Observed mass
Cd47	Cd47_1	+A+G+CdTdGdAdAd5Cd5CdGd5CdAdG+C+A+G	5379.4	5378.6
	Cd47_2	+A+C+GdGdAd5CdGdAdTdGd5CdAdA+G+G+G	5431.4	5430.6
	Cd47_3	+C+C+GdTdGd5CdGdGdTdTdTdTdT+C+A+G	5344.3	5343.5
	Cd47_4	+G+T+Gd5CdTdTdGdGd5CdGdAdGdT+C+T+C	5369.3	5368.5
Cd98	Cd98_1	+G+G+CdTdGd5CdTdTd5CdTd5Cd5CdG+G+T+T	5334.4	5333.6
	Cd98_2	+C+C+GdTd5Cd5CdGd5CdTdGd5CdAdT+T+C+A	5300.4	5299.7
	Cd98_3	+G+G+CdTdGd5Cd5CdAdGdTdGdGd5C+A+T+T	5378.4	5377.5
	Cd98_4	+G+C+AdGd5CdTdGdGdTdAdGdAdG+T+C+G	5413.3	5412.6
	Cd98_5	+G+C+AdAd5CdAdGdAdGdAdGd5CdG+C+T+C	5379.4	5378.7
	Cd98_6	+A+C+CdGdGd5Cd5Cd5CdGdAdAdTd5C+T+C+G	5334.4	5333.7
Mfap4	Mfap4_1	+G+C+AdTd5CdAdGdTdGdGd5CdAdG+G+G+C	5412.4	5411.6
	Mfap4_2	+C+T+GdAdTdAdGd5Cd5Cd5CdTdGdG+G+C+G	5377.4	5376.6
	Mfap4_3	+T+T+Cd5CdAdAdGdTd5Cd5CdAd5CdG+C+G+C	5309.4	5308.7
	Mfap4_4	+C+C+AdTdTdGdGdGd5Cd5Cd5CdAdA+T+T+G	5336.4	5335.6
	Mfap4_5	+C+T+Cd5CdGdTdGd5CdGd5CdTdTdG+A+G+G	5368.4	5367.6
	Mfap4_6	+T+G+CdTdGdAd5CdAdGd5Cd5CdTdA+G+C+C	5335.4	5334.6
	Mfap4_7	+G+G+TdGdGdGdGdTdGd5CdAdAdA+A+G+C	5448.4	5447.6
	Mfap4_8	+A+C+AdGdGdTdGd5CdTdGd5CdAdT+G+A+C	5371.4	5370.6
	Mfap4_9	+T+G+Gd5CdAdAdTdGd5Cd5CdAd5CdA+G+G+T	5371.4	5370.6

Supplementary Table S1. Gapmer ASOs used in Supplementary Figure S3. dN = DNA, +N = LNA, uN = 2′-*O*-MOE, d5C = 5-methyl-deoxycytosine. All internucleotide linkages were phosphorothioate.

Figure 1A

Gene	Technical replicate	PBS					2′-*O*-MOE Malat1_1					LNA Malat1_2				
		1	2	3	4	5	1	2	3	4	5	1	2	3	4	5
Ppib	a	24.16	24.05	24.40	23.85	24.49	25.54	24.80	25.96	25.28	25.65	25.36	26.02	25.47	25.89	24.94
	b	24.20	24.22	24.66	23.88	24.78	25.57	24.82	25.93	25.07	25.52	25.29	25.95	25.46	25.90	24.93
Malat1	a	19.15	18.53	18.79	18.25	18.40	21.11	21.01	21.51	20.32	21.48	22.69	22.98	22.85	23.27	21.37
	b	18.88	18.37	18.61	18.29	18.45	21.10	20.85	21.45	20.31	21.49	22.64	22.98	23.11	23.41	21.50

Figure 1B

Tissue	Gene	Technical replicate	PBS					Malat1_3				
			1	2	3	4	5	1	2	3	4	5
Bronchus	*Ppib*	A	22.43	22.38	21.96	22.60	22.36	23.01	22.21	22.82	22.35	22.64
		B	22.03	22.06	21.67	22.29	21.91	22.63	21.93	22.50	22.11	22.46
	Malat1	a	16.40	16.50	16.70	17.07	17.30	19.03	18.18	18.07	17.55	18.18
		b	16.27	16.28	16.52	16.89	17.02	18.69	18.03	17.91	17.38	18.22
Left lobe (proximal)	*Ppib*	a	22.62	23.08	22.28	22.43	22.30	22.35	22.10	22.53	22.92	22.14
		b	22.13	22.29	21.93	22.04	22.04	22.02	22.08	22.51	22.57	22.39
	Malat1	a	15.64	15.90	15.63	15.49	15.84	17.54	16.94	17.97	18.07	17.64
		b	15.49	15.61	15.21	15.46	15.44	17.69	17.12	17.69	17.97	17.65
Inferior lobe (distal)	*Ppib*	a	23.43	24.13	23.42	23.51	23.68	24.84	24.53	24.62	23.94	24.80
		b	23.20	23.88	23.32	23.44	23.40	24.43	24.25	24.33	23.85	24.73
	Malat1	a	17.69	18.43	17.29	17.16	17.25	20.78	19.70	20.46	20.20	20.92
		b	17.15	17.96	17.23	17.16	17.21	20.44	19.55	20.36	20.05	20.33

Figure 1C

Tissue	Gene	Technical replicate	PBS						NTC_1						Malat1_3					
			1	2	3	4	5	6	1	2	3	4	5	6	1	2	3	4	5	6
Lung	*Ppib*	a	24.12	24.28	23.83	23.56	24.18	23.44	25.09	24.68	24.33	24.94	24.52	24.64	24.34	24.23	24.08	24.52	24.53	24.52
		b	24.08	24.22	23.81	23.64	24.23	23.46	24.97	24.75	24.46	25.05	24.58	24.85	24.53	24.37	24.16	24.57	24.59	24.57
	Malat1	a	18.42	18.59	18.11	17.83	18.35	17.76	19.22	18.98	18.79	19.30	18.64	19.05	20.93	20.87	20.82	21.52	21.27	21.48
		b	18.67	18.62	18.26	18.03	18.49	17.98	19.33	19.13	18.91	19.46	18.83	19.13	21.28	21.15	21.12	21.70	21.45	21.78
Liver	*Ppib*	a	23.83	23.62	23.76	23.73	23.88	23.68	23.97	23.48	23.54	23.52	23.72	23.98	23.94	23.67	23.70	23.85	24.19	23.94
		b	24.04	23.85	23.62	24.03	23.99	23.83	24.05	23.53	23.61	23.47	23.56	23.86	23.83	23.59	23.65	23.92	24.13	23.96
	Malat1	a	20.27	20.13	20.20	20.26	20.32	20.26	20.24	20.21	20.13	20.12	20.23	20.40	21.19	21.47	20.90	21.14	21.43	21.39
		b	20.38	20.14	20.22	20.16	20.27	20.20	20.55	20.12	20.07	20.16	20.27	20.27	21.30	21.37	21.06	21.29	21.58	21.35
Kidney	*Ppib*	a	23.36	23.28	23.36	23.12	23.26	23.30	23.30	23.11	23.33	23.48	23.49	24.05	23.40	23.25	23.24	23.22	23.40	23.39
		b	23.48	23.31	23.28	23.18	23.22	23.53	23.30	23.10	23.24	23.47	23.39	23.88	23.26	23.21	23.18	23.29	23.22	23.35
	Malat1	a	18.06	18.15	18.16	18.01	18.14	18.22	18.16	18.10	18.05	18.41	18.37	18.75	18.47	18.65	18.64	18.58	18.58	18.73
		b	18.09	18.04	18.10	17.88	18.05	18.18	18.23	17.99	17.95	18.28	18.26	18.59	18.51	18.37	18.52	18.47	18.51	18.59
Spleen	*Ppib*	a	25.20	24.59	24.20	24.52	24.27	24.40	24.51	24.54	24.13	24.42	24.15	24.62	23.70	23.76	23.58	23.71	23.93	24.63
		b	25.12	24.60	24.27	24.48	24.41	24.34	24.47	24.49	24.00	24.54	24.25	24.55	23.79	23.88	23.68	23.79	23.85	24.68
	Malat1	a	20.00	19.35	19.29	19.13	18.80	19.01	18.67	18.72	18.59	19.05	18.93	19.24	18.58	18.61	18.55	18.56	19.13	19.35
		b	20.18	19.41	19.47	19.30	19.05	19.34	19.11	19.09	18.61	19.30	19.27	19.43	19.08	18.94	18.91	18.97	19.23	19.67

Supplementary Table S2. Raw Ct values of RT-qPCR analysis (Page 1/3).

Figure 3A

Time	Gene	Technical replicate	PBS						Cd47_4					
			1	2	3	4	5	6	1	2	3	4	5	6
1 week	*Ppib*	a	23.73	23.47	23.25	23.37	23.67	23.26	23.60	23.51	23.53	23.80	23.93	24.40
		b	23.88	23.54	23.21	23.45	23.67	23.49	23.64	23.58	23.44	23.89	24.06	24.44
	Cd47	a	22.14	21.79	21.28	21.56	21.87	21.23	22.04	22.26	22.26	22.86	23.03	23.37
		b	22.16	21.83	21.43	21.68	22.04	21.45	22.20	22.46	22.37	23.00	22.99	23.48
2 weeks	*Ppib*	a	23.00	23.91	23.33	23.72	24.12	23.53	24.11	24.60	23.36	24.28	25.08	25.01
		b	23.12	24.01	23.32	23.68	24.24	23.62	24.07	24.60	23.36	24.40	25.08	25.22
	Cd47	a	21.12	21.98	21.27	21.75	22.19	21.75	22.58	23.21	22.15	23.19	23.93	23.74
		b	21.32	22.30	21.55	22.02	22.58	22.17	22.96	23.42	22.45	23.49	24.11	23.88
4 weeks	*Ppib*	a	23.59	24.32	23.16	22.50	22.69		22.56	22.40	22.41	22.78	23.12	
		b	23.71	24.34	23.21	22.53	22.98		22.58	22.48	22.50	22.74	23.18	
	Cd47	a	21.61	22.13	21.18	20.69	20.84		21.03	21.36	21.37	21.21	22.13	
		b	21.77	22.29	21.35	20.89	21.04		21.23	21.42	21.86	21.26	22.06	
8 weeks	*Ppib*	a	23.11	22.82	23.50	23.61	22.43	22.82	22.59	22.39	22.53	22.57	23.02	23.94
		b	23.19	23.05	23.41	23.74	22.56	22.90	22.65	22.38	22.46	22.68	22.94	24.12
	Cd47	a	20.84	20.93	21.21	21.26	20.59	20.92	21.00	20.75	21.04	21.11	21.30	22.43
		b	21.21	21.09	21.47	21.42	21.05	21.20	21.19	21.03	21.36	21.36	21.58	22.50

Figure 4A

Gene	Technical replicate	PBS	Malat1_3
Ppib	a	23.22	23.61
	b	23.14	23.64
Malat1	a	17.52	19.30
	b	17.57	19.33

Supplementary Table S2–***Continued*****.** Raw Ct values of RT-qPCR analysis (Page 2/3).

Figure 5A

Gene	Technical replicate	PBS						Mfap4_4					
		1	2	3	4	5	6	1	2	3	4	5	6
Ppib	a	24.24	23.76	24.05	24.25	24.63	24.56	24.55	24.35	24.68	24.10	25.11	24.83
	b	24.07	23.70	23.84	24.07	24.51	24.37	24.59	24.30	24.68	24.13	25.12	24.71
Mfap4	a	22.53	21.64	22.51	22.15	22.53	22.92	26.34	25.66	26.37	25.39	26.47	26.13
	b	22.60	21.71	22.61	22.18	22.67	22.90	26.43	25.87	26.49	25.44	26.67	26.11

Figure 5B

qPCR Batch	Gene	Technical replicate	NTC_2 (10 nmol)					NTC_2 (20 nmol)					Adam33_1 (2.5 nmol)					Adam33_1 (5 nmol)					Adam33_1 (10 nmol)					Adam33_1 (20 nmol)				
			1	2	3	4	5	1	2	3	4	5	1	2	3	4	5	1	2	3	4	5	1	2	3	4	5	1	2	3	4	5
a	*Ppib*	a	24.32	24.13	23.84	24.31	24.31	24.17	24.20	24.07	24.28	24.60	24.21	24.21	23.78	23.34	24.48	24.62	24.28	23.91	23.88	24.62	24.01	23.23	23.90	23.12	22.81					
		b	24.51	24.06	23.69	24.36	24.18	24.25	24.13	24.11	24.33	24.52	24.12	24.16	23.55	23.26	24.34	24.67	24.23	23.62	24.07	24.70	24.05	23.36	24.02	23.21	23.02					
	Adam33	a	30.18	30.34	30.13	30.94	30.72	30.18	30.46	29.92	30.58	30.58	31.33	31.38	31.47	31.65	31.33	32.23	32.04	32.37	32.34	32.41	33.00	33.00	33.23	33.09	33.11					
		b	30.24	30.62	30.11	31.09	30.44	30.10	30.25	30.10	30.46	30.47	31.17	31.45	31.34	31.79	31.20	32.13	32.17	32.10	32.34	32.12	32.81	32.45	33.20	33.04	33.39					
b	*Ppib*	a	24.35	24.04	23.60	24.27	24.26																					22.98	23.37	23.23	24.50	23.41
		b	24.40	24.06	23.62	24.19	24.17																					23.12	23.22	23.20	24.47	23.43
	Adam33	a	30.83	30.57	30.68	31.56	31.34																					33.25	33.73	33.27	33.28	33.66
		b	30.85	31.03	30.82	31.45	31.44																					33.17	33.67	33.29	33.07	33.12

Figure 5C

Time	Gene	Technical replicate	NTC_2					Adam33_1				
			1	2	3	4	5	1	2	3	4	5
2 days	*Ppib*	a	25.37	25.27	25.09	24.91	24.55	25.06	24.44	24.41	24.36	24.83
		b	25.37	25.43	25.22	24.93	24.76	25.02	24.54	24.50	24.47	24.83
	Adam33	a	31.44	31.04	31.33	31.16	31.43	32.60	32.39	32.50	34.15	33.14
		b	31.32	31.05	31.40	31.15	31.20	32.71	32.79	32.27	33.35	33.23
1 Week	*Ppib*	a	24.27	23.99	23.66	24.22	24.22	23.97	23.21	23.95	23.27	23.12
		b	24.29	24.08	23.71	24.37	24.27	23.84	23.28	24.08	23.37	23.12
	Adam33	a	30.72	30.86	30.60	31.27	31.11	33.49	33.47	32.89	33.59	33.57
		b	30.56	30.76	30.66	31.56	30.97	33.04	33.15	33.38	33.48	33.30
2 Weeks	*Ppib*	a	24.42	23.89	23.88	23.89	24.25	24.39	24.84	24.29	24.22	25.26
		b	24.44	24.36	23.83	23.91	24.27	24.53	24.96	24.49	24.25	25.09
	Adam33	a	30.62	30.46	30.15	30.32	30.65	31.65	32.01	32.05	32.15	33.45
		b	30.47	30.73	30.50	30.31	30.43	32.16	32.08	32.18	31.95	33.02
3 Weeks	*Ppib*	a	23.90	23.57	24.83	24.48	24.36	25.09	25.06	24.71	24.20	25.11
		b	23.94	23.52	24.80	24.62	24.33	25.08	24.94	24.81	24.23	25.16
	Adam33	a	30.65	30.13	31.34	30.80	31.00	32.13	32.13	32.25	31.96	32.70
		b	30.56	30.24	31.34	30.90	30.92	32.19	32.07	32.22	31.88	32.62
4 Weesk	*Ppib*	a	25.40	25.97	25.92	25.01	25.47	25.47	26.34	24.50	24.96	25.67
		b	25.44	26.01	25.95	25.07	25.40	25.48	26.14	24.58	24.95	25.55
	Adam33	a	31.49	31.85	31.39	31.41	31.25	32.46	33.15	32.05	32.98	32.67
		b	31.48	32.07	31.23	31.17	31.06	32.48	32.73	32.50	32.56	32.66

Supplementary Figure S3A

Gene	Technical replicate	PBS			Cd47_1			Cd47_2			Cd47_3			Cd47_4		
		1	2	3	1	2	3	1	2	3	1	2	3	1	2	3
Ppib	a	24.04	23.57	23.52	24.02	24.30	24.47	24.91	24.57	24.19	24.43	24.07	24.30	24.22	24.24	24.28
	b	24.02	23.64	23.56	24.00	24.26	24.33	24.71	24.65	24.14	24.46	24.08	24.37	24.10	24.12	24.42
Cd47	a	21.68	21.29	21.52	21.93	22.23	22.22	22.93	23.05	22.53	22.49	22.17	22.41	22.46	22.30	22.89
	b	21.71	21.37	21.37	21.73	22.20	22.12	23.03	23.06	22.47	22.44	22.14	22.52	22.50	22.52	22.79

Supplementary Figure S3C

Gene	Technical replicate	PBS		Mfap4_1		Mfap4_2	Mfap4_3	Mfap4_4	Mfap4_5	Mfap4_6	Mfap4_7	Mfap4_8	Mfap4_9
		1	2	1	2	1	1	1	1	1	1	1	1
Ppib	a	25.13	25.12	24.56	24.14	24.18	24.31	25.08	25.15	24.23	24.20	24.99	25.15
	b	25.19	25.17	24.58	24.25	24.47	24.33	25.14	25.14	24.40	24.21	25.10	25.29
Mfap4	a	24.20	24.99	25.17	25.14	25.36	24.80	26.59	26.48	24.95	24.89	25.39	28.38
	b	24.15	24.93	25.13	25.09	25.23	24.74	26.69	26.49	24.73	24.83	25.23	28.27
Il6	a	35.26	34.45	34.22	32.37	31.98	34.22	31.97	35.18	33.80	31.07	31.31	ND
	b	35.82	34.71	34.48	33.00	32.48	35.39	31.95	35.96	34.14	31.16	31.64	30.27

Supplementary Table S2–*Continued***.** Raw Ct values of RT-qPCR analysis (Page 3/3).
